# The role of schools in the spread of mumps among unvaccinated children: a retrospective cohort study

**DOI:** 10.1186/1471-2334-11-227

**Published:** 2011-08-24

**Authors:** Wilhelmina LM Ruijs, Jeannine LA Hautvast, Reinier P Akkermans, Marlies EJL Hulscher, Koos van der Velden

**Affiliations:** 1 Department of Primary and Community Care, Academic Collaborative Centre AMPHI, Radboud University Nijmegen Medical Centre, Geert Grooteplein 21, 6525 EZ Nijmegen, The Netherlands; 2 Municipal Health Service GGD Rivierenland, J.S. de Jongplein 2, 4001 WG Tiel, The Netherlands; 3 Scientific Institute for Quality of Healthcare, Radboud University Nijmegen Medical Centre, Geert Grooteplein 21, 6525EZ Nijmegen, The Netherlands

## Abstract

**Background:**

In the Netherlands, epidemics of vaccine preventable diseases are largely confined to an orthodox protestant minority with religious objections to vaccination. The clustering of unvaccinated children in orthodox protestant schools can foster the spread of epidemics. School closure has nevertheless not been practiced up until now. A mumps epidemic in 2007-2008 gave us an opportunity to study the role of schools in the spread of a vaccine preventable disease in a village with low vaccination coverage.

**Methods:**

A retrospective cohort study was conducted among the students in four elementary schools and their siblings. The following information was collected for each child: having had the mumps or not and when, school, age, MMR vaccination status, household size, presence of high school students in the household, religious denomination, and home village. The spread of mumps among unvaccinated children was compared for the four schools in a Kaplan-Meier analysis using a log-rank test. Cox proportional hazard analyses were performed to test for the influence of other factors. To correct for confounding, a univariate Cox regression model with only school included as a determinant was compared to a multivariate regression model containing all possible confounders.

**Results:**

Out of 650 households with children at the schools, 54% completed a questionnaire, which provided information on 1191 children. For the unvaccinated children (N = 769), the Kaplan-Meier curves showed significant differences among the schools in their cumulative attack rates. After correction for confounding, the Cox regression analysis showed the hazard of mumps to be higher in one orthodox protestant school compared to the other (hazard ratio 1.43, p < 0.001). Household size independently influenced the hazard of mumps (hazard ratio 1.44, p < 0.005) with children in larger households running a greater risk.

**Conclusion:**

If and when unvaccinated children got mumps was determined by the particular school the children and their siblings attended, and by the household size. This finding suggests that school closure can influence the spread of an epidemic among orthodox protestant populations, provided that social distancing is adhered to as well. Further research on the effects of school closure on the final attack rate is nevertheless recommended.

## Background

In recent years, school closure has frequently been suggested as a strategy to mitigate epidemics. Using real life data on social contacts and serological evidence of infection, Wallinga et al. showed in a simulation study of the spread of mumps and pandemic influenza that school-aged children and young adults have the highest incidence of infection and contribute most to the further spread of infection during a respiratory epidemic in a completely susceptible population. This pattern is irrespective of the infectivity of the disease and suggests that the targeting of school-aged children to contain an epidemic can be very effective [1]. In addition, there are reports of the beneficial effects of school holidays and school strikes on the spread of influenza and other respiratory infections [2,3]. The exact role of schools in the spread of epidemics remains to be seen, however.

In the Netherlands, epidemics of vaccine preventable diseases are largely confined to the orthodox protestant minority population that objects to vaccination [4-6]. In the pluriform Dutch school system, moreover, orthodox Protestants have their own schools. There are about 125 orthodox protestant elementary schools and 7 orthodox protestant high schools, with the latter serving students from a large geographic region. In contrast to -- for example -- Belgium and the USA [7,8], vaccination is neither obligatory nor inquired about for school admission. The clustering of unvaccinated students in orthodox protestant schools may thus foster the spread of vaccine preventable diseases among this population, but school closure has yet to be practiced because it is assumed that the children will have considerable contact outside the school and infections can be transmitted during leisure time activities as well, especially in such a densely populated country as the Netherlands.

A mumps epidemic in the Netherlands in 2007-2008 [9] allowed us to conduct a retrospective cohort study of the role of schools in the spread of mumps in a village with low vaccination coverage. Mumps used to be a common childhood disease in the Netherlands, but the incidence decreased sharply after MMR vaccination was included in the National Immunization Program in 1987 [10,11]. MMR vaccination coverage in the Netherlands is high with over 95% for the first dose at age 14 months and over 90% for the second dose at age 9 years [12]. As already noted, MMR vaccination coverage is considerably lower among orthodox protestant groups with only about 55% and considerable variation across denominations from less than 15% to more than 85% [13]. In the autumn of 2007, a mumps epidemic occurred in the so-called Bible belt of the Netherlands where orthodox protestant groups live. The first cases were detected in the Rivierenland region [14] but, at the time, mumps was not a notifiable disease; general practitioners and pediatricians only reported laboratory confirmed cases on a voluntary basis. During the ensuing epidemic, at least 89 cases of mumps were reported to the National Institute of Public Health and the Environment [*RIVM*] [9]; 22 cases came from the Rivierenland region. As only a small minority of suspected cases underwent laboratory testing, the real extent of the epidemic was much larger than reported to the RIVM [15].

As suggested after previous epidemics of vaccine preventable diseases in the Netherlands [16], orthodox protestant schools may play a role in the spread of the disease. However, household contacts may play an even more important role than school contacts as household contact -- particularly in larger families -- is known to play a central role in the transmission of infectious diseases [17,18]. Orthodox protestant families generally refrain from family planning and are therefore usually large. In addition, other social contacts including the church may possibly play a role. For orthodox Protestants, the church is an important part of their social lives. They go to church twice on Sunday, and activities are often organized by the churches for children and young people. The spread of an epidemic along the lines of a religious denomination also thus seems plausible.

The aim of the present study was to assess the role of elementary schools in the spread of mumps among unvaccinated children in a village with low vaccination coverage due to religious objections. Research questions were if there are any differences in the attack rates and time of onset for the mumps among the unvaccinated children connected to the particular elementary schools. And if differences are detected, can they be explained by factors other than the school, such as the size of the household or the particular religious denomination.

## Methods

### Study design and population

We performed a retrospective cohort study in a village of 6000 inhabitants in the Rivierenland region of the Netherlands, which is in the middle of the Dutch Bible belt. In 2007, MMR vaccination coverage among the 9-year olds in this village was 44%. The village has 4 elementary schools, 2 of which are orthodox protestant, 1 protestant, and 1 public. An orthodox protestant high school as well as other high schools are in the neighboring towns. The study population consisted of all students in the four elementary schools and their siblings up to 21 years of age. The study period was from the 1^st ^of September 2007 to the 1^st ^of September 2008.

### Variables and data collection

For every child, the following determinants were collected: elementary school connection (orthodox protestant schools A and B, other elementary schools C and D), age (in years), MMR vaccination status (no MMR, 1 MMR, or 2 MMR), household size (≤ 3 or > 3 children), presence of high school students in the household (yes or no), denomination (Reformed Congregations, Reformed Congregations in the Netherlands, other protestant denomination, other or no religion), and home village (study village or other village). Outcome variables were clinical signs of mumps (yes or no) as assessed by the parents and the week of onset for the clinical signs of mumps.

Based on the WHO clinical case definition mumps was defined as an acute onset of swelling of the cheeks lasting at least two days [19]. The week of onset of clinical signs of mumps was measured with respect to the start of the epidemic in the village as defined by the regional health authorities. The week of onset also thus represents the survival time until the mumps appeared.

Questionnaires were distributed via the four elementary schools. The 650 households with one or more children attending one of these schools were invited to participate in the study. The parents received an introductory letter from the municipal health service, which explained the aims of the study and offered to provide additional information. The recipients were asked to complete one questionnaire per household and thereby provide information on all of the individuals up to 21 years living in the household. Given the sensitive nature of the topic of vaccination in the orthodox protestant minority population, the questionnaires were completed anonymously and the vaccination data provided by the parents were not checked against the national vaccination register. The completed questionnaires were returned to the municipal health service via regular mail, free of charge. Return of the questionnaire was considered informed consent.

### Analysis

Possible differences in the characteristics of the households and children in the four elementary schools were tested by ANOVA or chi-square tests.

The spread of mumps among the unvaccinated children --in terms of cumulative attack rate over time-- was compared for the four schools by Kaplan-Meier-analysis, using a log-rank test. A Cox proportional hazard analysis was performed to examine the influence of other factors like household size and religious denomination. Due to the small numbers of unvaccinated children in schools C and D (i.e., 28 and 3, respectively), the Cox proportional hazard analysis was restricted to the orthodox protestant schools A and B. To correct for confounding, a univariate Cox regression model containing only school connection as determinant was compared to a multivariate model containing all possible confounders (age, household size, presence of high school students in the household, religious denomination, and home village). Statistical analyses were performed using SPSS version 16.0. A p-value < 0.05 was considered significant.

### Ethics

The research conformed to the Helsinki declaration and Dutch legislation and was approved by the research ethics committee of the Radboud University Nijmegen Medical Centre, reference number 2010/431.

## Results

### Response and characteristics of the study population

Of the 650 households with one or more children attending one of the villages' elementary schools, 54% (351) completed the questionnaire. This provided information on 1191 children 0 to 21 years of age. The characteristics of the respondents per school are shown in Table 1. Vaccination coverage varied widely across the schools: From less than 15% for those children with a connection to the orthodox protestant schools to over 90% for those children with a connection to the other schools.

**Table 1:** Response rates, household characteristics, and child characteristics per school

School	Response house-holds (%)	Households with secondary school **pupils**^**1**^ (%)	Mean number of children **per household**^**2**^ (range)	Children in study	**Mean age**^**3**^ (SD)	Children living outside study **village**^**4**^ (%)	**MMR vaccination status**^**5**^		
							
							Unvaccinated (%)	MMR1 (%)	MMR2 (%)
A	124 (55)	68 (55)	4,1 (1-12)	504	10,3 (± 5,3)	220 (44)	443 (88)	31 (6)	30 (6)

B	93 (50)	37 (40)	3,7 (1- 9)	344	9,3 (± 5,4)	110 (34)	295 (86)	31 (9)	18 (5)

C	97 (63)	48 (49)	2,8 (1- 7)	272	9,7 (± 4,9)	18 (7)	28 (10)	109 (40)	135 (50)

D	37 (37)	9 (24)	2,0 (1- 4)	71	9,5 (± 4,8)	0 (0)	3 (4)	36 (51)	32 (45)

Total	351 (54)	162 (46)	3,3 (1-12)	1191	9,8 (± 5,2)	348 (29)	769 (65)	207 (17)	215 (18)

^1 ^Chi square 12.809, df 3, p = 0.005

^2 ^ANOVA: Welch F = 43.983, df 168.178, p < 0.005

^3^ANOVA: F = 2.566, df 3, p = 0.053

^4 ^Chi-square 1.490 E2, df3, p < 0.005

^5^Chi-square 6.578 E2, df6, p < 0.005

The children with a connection to the orthodox protestant schools A and B belonged to largely two orthodox protestant denominations, namely the Reformed Congregations and the Reformed Congregations in the Netherlands. Both denominations were represented at both of the schools. The children with a connection to school C belonged to largely the other protestant denominations. The majority of the children connected to school D had some other or no religious denomination.

### Spread of mumps in relation to the four schools

Almost half of the respondents (47%, 95% CI 45-50%) reported clinical signs of mumps.

The vast majority (98%, 95% CI 96-99%) of the cases occurred among the unvaccinated children. The attack rates across the four schools varied widely (see Table 2), which could be expected in light of the major differences in vaccination coverage. For the subgroup of unvaccinated children, the attack rates also varied across the schools with the rates much higher for those children with a connection to the orthodox protestant schools A and B than for those children with a connection to schools C and D (p < 0.05)(see Table 2). Furthermore, 59% (109/186) of the cases among the students at school A and 53% (68/128) of the cases among the students at school B could be classified as possibly secondary cases of mumps (i.e., onset of symptoms one incubation period past the infectious period of another case in the same grade, thus in the third week following onset of symptoms of the other case). Using the same definition, there were no possibly secondary cases at schools C and D.

**Table 2:** Mumps attack rate per school for all children and for unvaccinated children

	All children			Unvaccinated children		
**School**	**N**	**Attack** ** rate (%)**	**95% CI**	**N**	**Attack** ** rate (%)**	**95% CI**

A	504	66	62-70	443	75	71-79

B	344	63	58-68	295	72	67-77

C	272	5	3- 8	28	32	15-49

D	71	1	0- 4	3	0	-

Total	1191	47	45-50	769	72	

**Figure 1:**
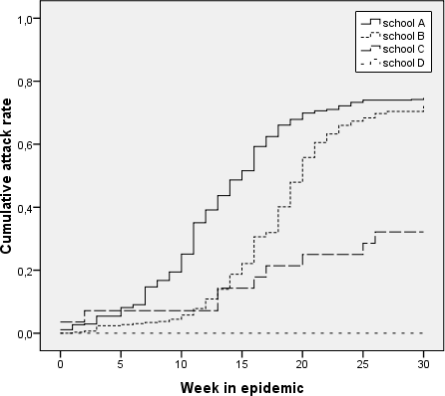
**Spread of mumps among unvaccinated children connected to four elementary schools**.

The Kaplan-Meier curves showed significant differences in the cumulative attack rates over time for the four elementary schools (log rank test p < 0.001, see Figure 1). The epidemic affected unvaccinated children with a connection to school A significantly earlier than unvaccinated children with a connection to school B (log rank test p < 0.05). For the three unvaccinated children with a connection to school D no cases of mumps were reported.

### Comparison between the two orthodox protestant schools

In order to check that the significant differences in the Kaplan-Meier curves were caused by the connection to the particular elementary schools and not by such factors as household size or religious denomination, Cox proportional hazard analyses were performed. Due to the small numbers of unvaccinated children with connections to schools C and D, these analyses were restricted to the orthodox protestant schools A and B.

The univariate Cox proportional hazard analysis showed the hazard of mumps among the unvaccinated children with a connection to school A to be significantly higher than the hazard among the unvaccinated children with a connection to school B. The hazard ratio was 1.45 (95% CI 1.22-1.72).

Multivariate Cox proportional hazard analyses showed no confounding by age, household size, presence of high school students in the family, religious denomination, or home village. The corrected hazard ratio for school A compared to school B was 1.43 (95% CI 1.19-1.71), see Table 3. In addition to the school connection, however, the household size appeared to independently influence the risk of getting the mumps, with a hazard ratio of 1.44 (p < 0.005) for unvaccinated children from large households (> 3 children) versus unvaccinated children from small households, see Table 3.

**Table 3:** Hazard ratios for possible determinants of mumps in unvaccinated children related to orthodox protestant schools

	Hazard ratio	95% CI	p
**School**^**1**^	1.43	1.19-1.71	< 0.001

**Age**^**2**^	1.00	0.98-1.16	0.97

**Household size**^**3**^	1.44	1.16-1.79	< 0.005

**High school students** **in household**^**4**^	1.13	0.89-1.42	0.32

**Denomination**			0.39
**- *Reformed Congregations***^**5**^	0.77	0.53-1.13	0.18
**- *Reformed Congregations*** ***in the Netherlands***^**6**^	0.79	0.56-1.12	0.19

**Home village**^**7**^	1.16	0.96-1.40	0.13

^1 ^School A compared to school B

^2 ^Age in years

^3 ^Families with > 3 children compared to families with ≤ 3 children

^4 ^Families with high school students compared to families without high school students

^5 ^Reformed Congregations compared to other protestant denominations

^6 ^Reformed Congregations in the Netherlands compared other protestant denominations

^7 ^Other villages compared to study village

## Discussion

This retrospective cohort study shows the role of schools in the spread of an epidemic of mumps among unvaccinated children in a village with low vaccination coverage. When four schools in the same village were compared, the final mumps attack rates were much higher among the unvaccinated children with a connection to a orthodox protestant school than among the other unvaccinated children. Given that the vaccination coverage in the non-orthodox schools was above 90%, the low attack rates among the unvaccinated children with a connection to these schools can probably be attributed to a herd effect [20].

When the two orthodox protestant schools were compared, the unvaccinated children with a connection to orthodox protestant school A were affected earlier during the epidemic than the unvaccinated children with a connection to the other orthodox protestant school within the same village (i.e., school B). Given that we controlled for possibly confounding factors, this finding shows schools to play a role in the spread of infectious disease among orthodox protestant groups. Schools involve social clustering and, once the mumps has been introduced into a school, it can thus spread more easily among children at the same school than among other children. The school attended by unvaccinated children and their siblings -- together with household size -- thus determined whether the children got the mumps or not and when. And this suggests that school closure can influence the spread of an epidemic within an orthodox protestant population.

The question, of course, is whether or not school closure influences the final outcome of the epidemic. In a simulation study of pandemic influenza, the closing of schools and keeping children at home reduced the final attack rate by 90% -- without the further use of vaccines or antivirals. For this result, however, the children had to be quarantined for the extent of the epidemic, which is given the impact on education not desirable and obviously not achievable in real life [21].

When considering the effects of school closure, compliance with social distancing during school closure is of critical importance. Recent experiences with school closure for influenza prevention showed the majority of the children to visit at least one social event during the school closure period [22,23]. Nevertheless, over all contact rates during a school closure period are likely to be considerably lower than during regular school periods. German school children reported four times less contacts on Sundays than on school days, for example [24].

According to an international diary study about 20% of the contact for people living in the Netherlands is leisure time contact e.g., during sports or other activities [25]. The orthodox protestant way-of-life differs greatly from this, however. For religious reasons, members of this population refrain from sports, cinema, and television [26]. Leisure time activities are nevertheless organized by the churches for such orthodox protestant children, which means that the variable religious denomination can serve as a proxy variable for leisure time activities. In the present study, religious denomination was nevertheless not found to significantly influence the spread of mumps. However, orthodox protestant children will --like other children-- visit family and friends. In the extra leisure time generated by school closure social distancing remains therefore of critical importance.

The perceived seriousness of a disease is an important determinant of compliance with social distancing [22]. According to another study that we conducted, orthodox protestant parents perceive polio to be a particularly serious health threat and thus something that warrants not only social distancing but even consideration of vaccination (manuscript in preparation). Schools may also play a role in the spread of polio. At the beginning of the 1992-1993 polio epidemic, laboratory signs of polio infection were far more prevalent at the orthodox protestant schools of the siblings of the index case than at other schools [27]. Therefore we recommend that school closure be considered during a next polio outbreak. We further recommend additional research and simulation studies in particular to gain more insight into the effects of school closure on the final attack rates of epidemics of vaccine preventable diseases in orthodox protestant populations, while also taking the durations of school closure and levels of vaccination coverage into account.

### Some possible limitations on the present study

The overall response rate in our study was 54%. The response rates at the orthodox protestant schools A and B were slightly higher than the response rate of 48% in another study among a orthodox protestant population [28] and considerably higher than the response rate of 37% at school D where mumps did not appear to be an issue. Given that school D was the smallest school in the village and -- as a public school -- had a nationally representative vaccination coverage of > 95%, we do not think that the low response rate of this school affected our results. A non-response analysis was nevertheless not feasible as vaccination is a sensitive subject and the respondents in our study returned their questionnaires anonymously; we could not, thus, check the actual vaccination status of our respondents in the national register.

Several reports indicate that parental recall of vaccination may be inaccurate but that the inaccuracy concerns mostly the number of injections and vaccination dates [29-31]. Given that vaccination is a particularly sensitive topic among orthodox Protestants, we expected our respondents to recall at least whether or not their children are vaccinated against MMR. Nevertheless, recall inaccuracy is a possible limitation on the present study.

When the vaccination coverage of 35% among our respondents is compared to the registered vaccination coverage for the village (44%), unvaccinated respondents appear to be overrepresented. This overrepresentation can be explained, however, by the participation of students from orthodox protestant schools and their siblings who *live *in other villages.

The outcome variable in the present study was the clinical diagnosis of mumps. As mumps is generally construed to be a mild disease, only a minority of patients consult their GPs with regard to symptoms. Our case definition was therefore based upon clinical assessment by the parents while it is known that 30% of cases of mumps infection go without symptoms [32]. The real amount of mumps may therefore be underestimated in the present study, but such underestimation should apply to all schools and therefore not affect our comparison of the schools.

In closing it should be noted that as part of our recruitment strategy, households with only children under four years of age or only high school students were not included in the study. In orthodox protestant families, mothers are supposed to stay at home to care for their children, which means that transmission via day care centers that are rarely frequented by orthodox Protestants is not very likely. Transmission among high school students may, however, be more important in the spread of the epidemic, particularly during the early stages [1]. While the presence of high school students in a family with elementary school children did not influence the hazard of mumps, we cannot exclude the possibility that high school students played a role in the initial introduction of mumps into the village.

## Conclusion

During the mumps epidemic of 2007-2008 we studied the spread of mumps among unvaccinated children in a Dutch village with a large orthodox protestant population.

The particular school that was attended by the unvaccinated children and their siblings determined -- together with the size of the household -- whether these children got the mumps and when.

This suggests that school closure can influence the spread of future epidemics -- particularly in orthodox protestant populations and when social distancing is adhered to. Before deciding on school closure, however, further research is recommended to gain greater insight into the necessary duration of such school closure and its effects on final attack rates.

## Competing interests

The authors declare that they have no competing interests.

## Authors' contributions

WLMR conceived the study, participated in its design, collected the data, conducted the statistical analyses, and drafted the current article. JLAH participated in the design of the study, helped with the interpretation of the data and helped draft this article. RPA helped conduct the statistical analyses, helped with the interpretation of the data, and helped revise the present article. MEJL participated in the design of the study, helped with the interpretation of the data, and helped draft the present article. KvdV participated in the design of the study and helped revise the present article. All of the authors have read and approved the final manuscript.

## Authors' information

WLMR is preparing a thesis on "Acceptance of vaccination in orthodox protestant groups."

The mumps epidemic of 2007-2008 provided the opportunity to add a study on the spread of a vaccine preventable disease within a minority group with traditionally low vaccination coverage. The focus of the other research is on vaccination coverage within the various orthodox protestant denominations and the nature of the individual decisions made by the members of these denominations with regard to whether or not to vaccinate their children.

## Pre-publication history

The pre-publication history for this paper can be accessed here:

http://www.biomedcentral.com/1471-2334/11/227/prepub
